# The effect of low-frequency high-intensity ultrasound combined with aspirin on tooth movement in rats

**DOI:** 10.1186/s12903-023-03359-3

**Published:** 2023-09-05

**Authors:** Jiao Xin, Xinxin Zhan, Fu Zheng, Huazhi Li, Yixiang Wang, Cuiying Li, Jiuhui Jiang

**Affiliations:** 1 Central Laboratory, Peking University School and Hospital of Stomatology & National Center of Stomatology & National Clinical Research Center for Oral Diseases & National Engineering Research Center of Oral Biomaterials and Digital Medical Devices, Beijing, China; 2 Department of Orthodontics, Peking University School and Hospital of Stomatology & National Center of Stomatology & National Clinical Research Center for Oral Diseases & National Engineering Research Center of Oral Biomaterials and Digital Medical Devices, Beijing, China; 3 Central Laboratory, Department of Oral and Maxillofacial Surgery, Hospital of Stomatology & National Center of Stomatology & National Clinical Research Center for Oral Diseases & National Engineering Research Center of Oral Biomaterials and Digital Medical Devices, Peking University School, Beijing, China

**Keywords:** Aspirin, Orthodontic, Tooth movement, Ultrasound

## Abstract

**Background:**

Given the difficulties or incapacity of teeth movement in orthodontic treatment, the ways to speed tooth movement must be investigated. Besides, nonsteroidal anti-inflammatory drugs (NSAIDs) were utilized to treat pain caused by tooth movement during orthodontic treatment. The purpose of this study is to examine the impact of aspirin and low-frequency high-intensity ultrasound (LFHIU) on rat orthodontic tooth movement in rats.

**Methods:**

Thirty-six male Sprague-Dawley rats were divided into three groups: orthodontic (O), ultrasound-treated orthodontic (OU), and ultrasound-treated orthodontic with aspirin gavage (OUA) group. In the OU and OUA group, LFHIU (44 W/cm2, 28 kHz) was applied to the buccal side of the maxillary first molar alveolar bone for 10 s every day. In the OUA group, aspirin was given by gavage every day. The rats were sacrificed on days 1, 3, 7, and 14.

**Results:**

After ultrasonic treatment, the speed of tooth movement was increased by about 1.5 times. And the number of osteoclasts considerably increased by about 2 times. However, they decreased slightly after aspirin gavage. By Applying ultrasound therapy, Receptor Activator for Nuclear Factor-κ B Ligand (RANKL) levels in periodontal tissue were elevated. Aspirin was able to reduce these increases. Results from Micro Computed Tomography (Micro-CT) revealed that bone mineral density decreased by about 1/5 after ultrasound treatment on the compression side. The rate of bone mineral apposition indicated that bone was forming under tension, and that of the OU group increased by about 1.3 times that O group.

**Conclusions:**

Although aspirin slowed this trend, LFHIU still enhanced overall tooth mobility in orthodontic treatment.

## Background

Orthodontic treatment is becoming more popular due to the improvements it produces in masticatory function and aesthetics. However, because of the longer treatment duration [1] and the complications such as white spot lesions [2], dental root resorption [3], and periodontal disease [4] etc., researchers paid more attention to these concerns. Among them, it is a challenging issue that accelerating tooth movement or accelerating the movement of difficult-to-move teeth. Furthermore, it is a requirement for patients and a goal of clinicians that accelerate tooth movement healthily [5].

At present, methods of accelerating orthodontic teeth movement include surgical treatment, pharmaceutical treatment, and physical treatment, such as corticotomy [6], local injection of parathyroid hormone [7] or prostaglandin E2 [8], low-energy laser irradiation [9], vibration [10], and low-intensity pulsed ultrasound [11–14], etc. Trauma and side effects are the main concerns when using surgery or drugs [15–17]. Vibration [18], on the other hand, is a non-invasive treatment for accelerating tooth movement that has no notable adverse effects. Teruko et al. [19] verified that high-frequency vibration (3gF, 70 Hz, 3 min once a week) might increase osteoclastogenesis via NF-κB activation and hence speed tooth movement. Ultrasound [20], a non-invasive method like vibration, is also considered an effective way to promote tooth movement. Xue et al. [21] found that LIPUS (30 mW/cm^2^, 1.5 MHz, 20 min once a day) increased orthodontic teeth movement via the RANKL and HGF/Runx2/BMP-2 signaling pathways.

Previous researches showed low-intensity pulse ultrasound could speed up orthodontic tooth movement [11, 12, 22]. However, a clinical study [23] found that a single dose of LIPUS applied at 3 weeks did not speed up or lessen the pain associated with orthodontic tooth movement. Moreover, we could find that the treatment duration was around 20 min [24], which might reduce patients’ compliance. There was a study [25] showed that higher ultrasound intensity can make the RANKL gene expression higher at 24 h. One study examined the effects of ultrasonic at 1 MHz, 0.1 W/cm^2^, and 45 kHz, 0.03 W/cm^2^ on mesenchymal stem cells. It was discovered that 45 kHz ultrasonic vibration produced more prostaglandin E2 than 1 MHz ultrasonic [26]. Prostaglandin E2 increases the production of osteoclasts, implying that ultrasonic with a lower frequency may be more favorable to the formation of osteoclasts. Currently, almost all research on the acceleration of orthodontic teeth movement by ultrasonic is limited to low-intensity pulsed ultrasound and does not apply to higher intensity or lower frequency ultrasonic vibration.

Aspirin could inhibit osteoclast differentiation by activating receptor activators of the nuclear factor kappa-B ligand (RANKL/RANK) signaling pathway [27]. In addition, pain during orthodontic treatment has become an important reason for people to give up treatment [28]. Some literature studies have shown that non-steroidal anti-inflammatory drug, such as aspirin, was often used to reduce pain by orthodontic patients. However, researches showed most non-steroidal anti-inflammatory drugs might reduce tooth movement [29–32].

Therefore, the purpose of our study is to determine how orthodontic tooth movement in rats is affected by low-frequency high-intensity ultrasound (LFHIU) combined with aspirin, and whether it affects the RANKL/RANK signaling pathway.

## Methods

### Experimental animals

**Fig. 1:**
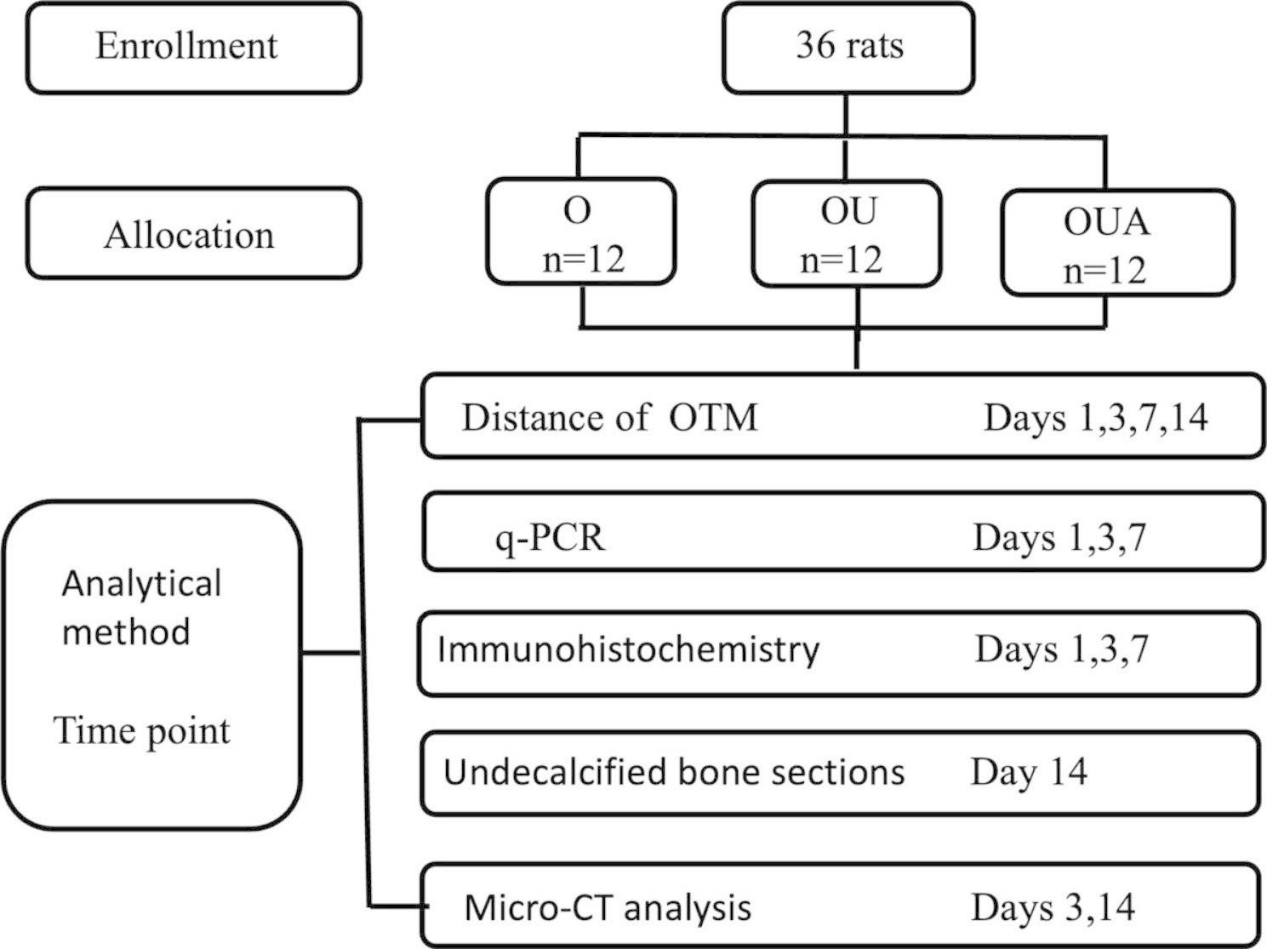
Flow diagram of the experiment

Thirty-six male Sprague-Dawley rats aged 6–8 weeks and weighing 160–180 g (provide by the Laboratory animal center of Peking University School and Hospital of Stomatology) were randomly divided into three groups (Fig. 1), orthodontic (O) group, ultrasound-treated orthodontic (OU) group, ultrasound-treated orthodontic with 100 mg/kg/day aspirin gavage (OUA) group. The sample size calculation referred to the previous literature [33], and the calculation formula is 2*SD^2^ (1.96 + 0.842) ^2^/d^2^. The rats were maintained at a controlled temperature (22 ± 2 °C), with 12-h light/12-h dark periods.

To avoid dislodgement, we prepared a retention groove at the middle of the anterior teeth and the first molar teeth crown, binding the two anterior teeth together. A force of 25 g measured with a force gauge (3 M Unitek, USA) was applied to the maxillary first molar and incisors using a superelastic NiTi (Sentalloy, Islandia, NY, USA) closed coil spring. Dental adhesive resin (3 M, St Paul, Minn) was then used to assist retention. The appliance was checked once per day to ensure that it had not fallen off or fractured. In the OU and OUA group, a CS2 work tip of the piezosurgery system (Satelec Acteon, France) was applied every day to the buccal side of the alveolar bone of the first molar to provide ultrasonic (44 W/cm^2^, 28 kHz) for 10 s. The transducer used in this study was rectangular, with an area of 0.5 cm^2^. The O group was treated with a sham transducer that did not emit ultrasound. In the OUA group, 100 mg/kg body weight of aspirin (Sigma-Aldrich, USA) with 0.5% sodium carboxymethylcellulose-assisted solubilization was given by gavage every day. The rats in the O and OU groups received the same volume of saline solution.

### Measurement of tooth movement

The rats were killed by injecting an overdose of anesthetic (pentobarbital > 25 mg/kg) on the 1st, 3rd, 7th, and 14th days. Three rats were harvested at each point in time. After dissection of the maxillary region, impressions of the teeth and maxillae were obtained with the use of individual trays containing hydrophilic vinyl polysiloxane impression material (EXAFAST Injection Type, GC Co., Tokyo, Japan). The amount of tooth movement was evaluated by measuring the closest distance between the first and the second molars in the impression under a stereoscopic microscope (VH-7000; Keyence, Osaka, Japan).

### Micro-computed tomography (micro-CT) analysis

Rats were euthanized on days 3 and 14 to evaluate bone mineral density (BMD. An Inveon MM system (Siemens, Munich, Germany) was used for micro-CT analysis. Images were obtained at a pixel size of 8.82 μm, exposure time of 1500 ms, voltage of 80 kV, and current of 500 µA for each of the 360 rotational steps. Cross-sections were used for general viewing, but vertical sections were used for quantitative analysis. The vertical reference plane is the plane formed by the proximal and distant middle cusp of the first molars and the root tip of the tooth. The horizontal reference plane is located in the one-third of the root near the neck of the tooth and perpendicular to the vertical plane. The region (200 μm width ×200 μm thickness × 800 μm length) of alveolar bone adjacent to the neck of the distal palatal root was regarded as the compression side.

### Sequential fluorochrome labeling and histomorphometric analysis

Rats were injected with alizarin-3-methyliminodiacetic acid (30 mg/kg body weight, i.p.) on the second day and calcein (20 mg/kg body weight, i.p.) on the 11th day. Rats were euthanized on the 14th day. Cross sections were cut parallel to the occlusal surface and polished to 60–80 μm with an EXAKT cutting and grinding system (EXAKT Apparatebau, Germany). The distal alveolar bone of the mesial palatal root was used for analysis. The bone mineral apposition rate (MAR) was measured using Bioquant software (BioQuant, San Diego, CA, USA). The maximum distance between the red fluorescent line and the green fluorescent line on the photo was measured.

### Immunohistochemistry and TRAP staining

Following a predetermined schedule, the maxillary bone containing the maxillary first molar was dissected on days 1, 3, and 7. The specimens were immediately immersed in 4% paraformaldehyde for 48 h, then demineralized in 10% EDTA (pH = 7.4) for 6 weeks. After paraffin embedding, the specimens were sectioned (4 μm) in the mesial-distal direction of the first molar and prepared for immunohistochemical staining and TRAP staining.

The slides were deparaffinized, treated with 0.125% trypsin and 5 µg/ml proteinase K solution for 20 min at 37 °C for antigen retrieval, and treated with 3% hydrogen peroxide for 20 min at room temperature. The sections were then blocked with goat serum for 1 h at room temperature, and incubated with antibodies to RANKL (1:200; sc-52,950, Santa Cruz Biotechnology, Santa Cruz, CA, USA) overnight at 4 °C. They were then incubated with secondary antibody for 20 min. After washing with phosphate-buffered saline, the sections were developed with diaminobenzidine. Ultimately, hematoxylin was used for counterstaining.

TRAP staining was performed with a tartrate-resistant acid phosphatase kit (Sigma-Aldrich, St. Louis, MO, USA) according to the manufacturer’s instructions, and the slides were counterstained with hematoxylin. Cells containing more than 3 nuclei were considered osteoclasts.

### mRNA extraction and quantitative real-time polymerase chain reaction (Q-PCR)

The periodontal ligament and alveolar bone were extracted from the maxillary first molar on days 1, 3, and 7. The tissues were broken down with a tissue lyser (Qiagen, Germany). RNA was then isolated using Trizol reagent (Invitrogen, Grand Island, NY, USA). Complementary DNA was synthesized from 2 µg of total RNA using a commercially available kit (Thermo Fisher Scientific, USA). Q-PCR reactions were performed in a 20-µl mixture using a thermal cycling system (Applied Biosystems 7500 Real-time PCR System, ABI) under the following cycle conditions: 15 min at 95 ℃, 40 cycles of 15 s at 90 ℃, 30 s at 60 ℃ and 20 s at 72 ℃. Gene expression was normalized to GAPDH expression, expressed as 2^−ΔΔCt^ (baseline in the control group) = 1. The primer sequences used in these studies are designed by the BLAST in National Center for Biotechnology Information (NCBI). The q-PCR primers are listed ( Table 1).

**Table 1:** Primer sequences used for q-PCR

Gene	Forward primer	Reverse primer
*RANKL*	GCTCAGCCTTCGTGTCCAAG	CCGTCGATCAGTTGGCGC
*GAPDH*	TCTCCTGCGACTTCAACAGTG	CTCTTACTCCTTGGAGGCCAT

### Statistical analysis

Image-Pro Plus 6.0 was used for analyzing the mean optical density value of RANKL in periodontal ligament. Integrated optical density/area was regarded as mean optical density. The descriptive statistics of SPSS software (IBM Corporation Armonk, NY, USA) was used to analyze whether the data is normally distributed. Statistical analyses were performed using one-way analysis of variance to compare the differences in compliance with normality. Results with P < 0.05 were considered statistically significant.

## Results

### The distance of tooth movement

**Fig. 2:**
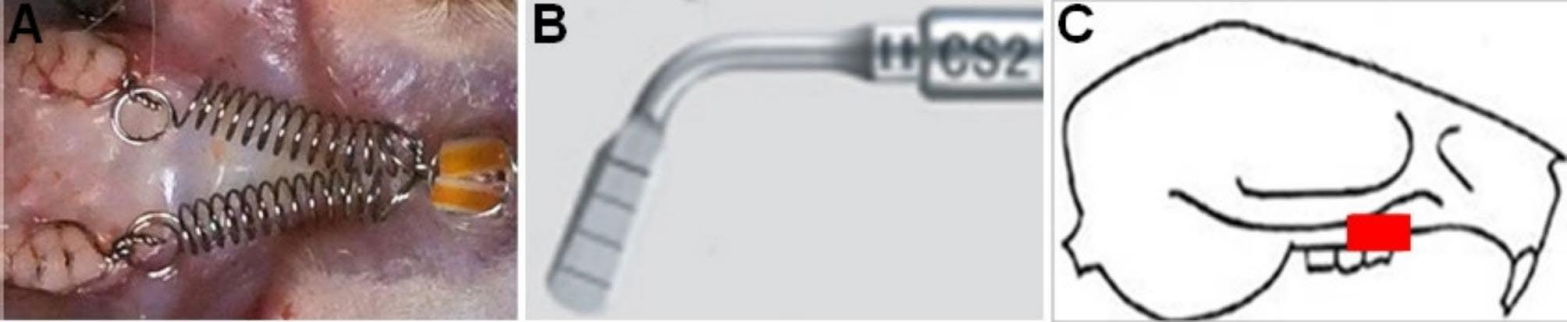
(**A**) Animal model of OTM. (**B**) Work tip of piezosurgery-CS2. (**C**) Location of application of ultrasonic vibration

**Fig. 3:**
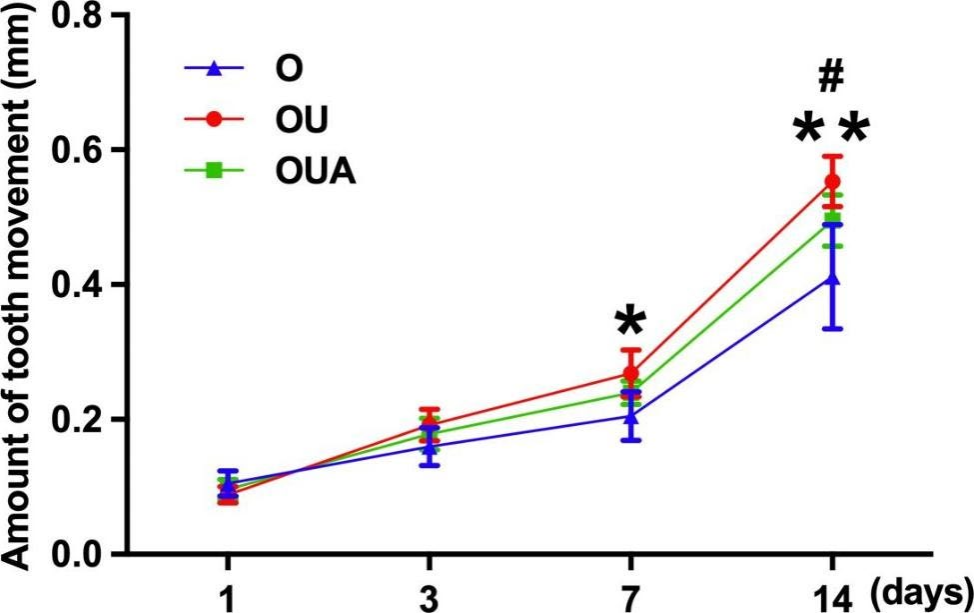
Distance of tooth movement. *P < 0.05, OU group versus O group; #p < 0.05, OU group versus OUA group; **P < 0.01, OU group versus O group

Figure 2 shows the experimental animal orthodontic force model and the location of ultrasonic stimulation. The difference in tooth movement distance across groups is compared by measuring the width of the impression after it is made, revealing the effect of ultrasound on tooth movement. The results (Fig. 3) showed that the distance of tooth movement was greatest in the OU group and least in the O group on the third, 7th, and 14th day, the difference between the two groups was statistically significant on the 7th and 14th day (P<0.05). As shown in Fig. 3, there was a significant difference between the OU and OUA groups on day 14 (P<0.05).

### Osteoclast response to ultrasound

**Fig. 4:**
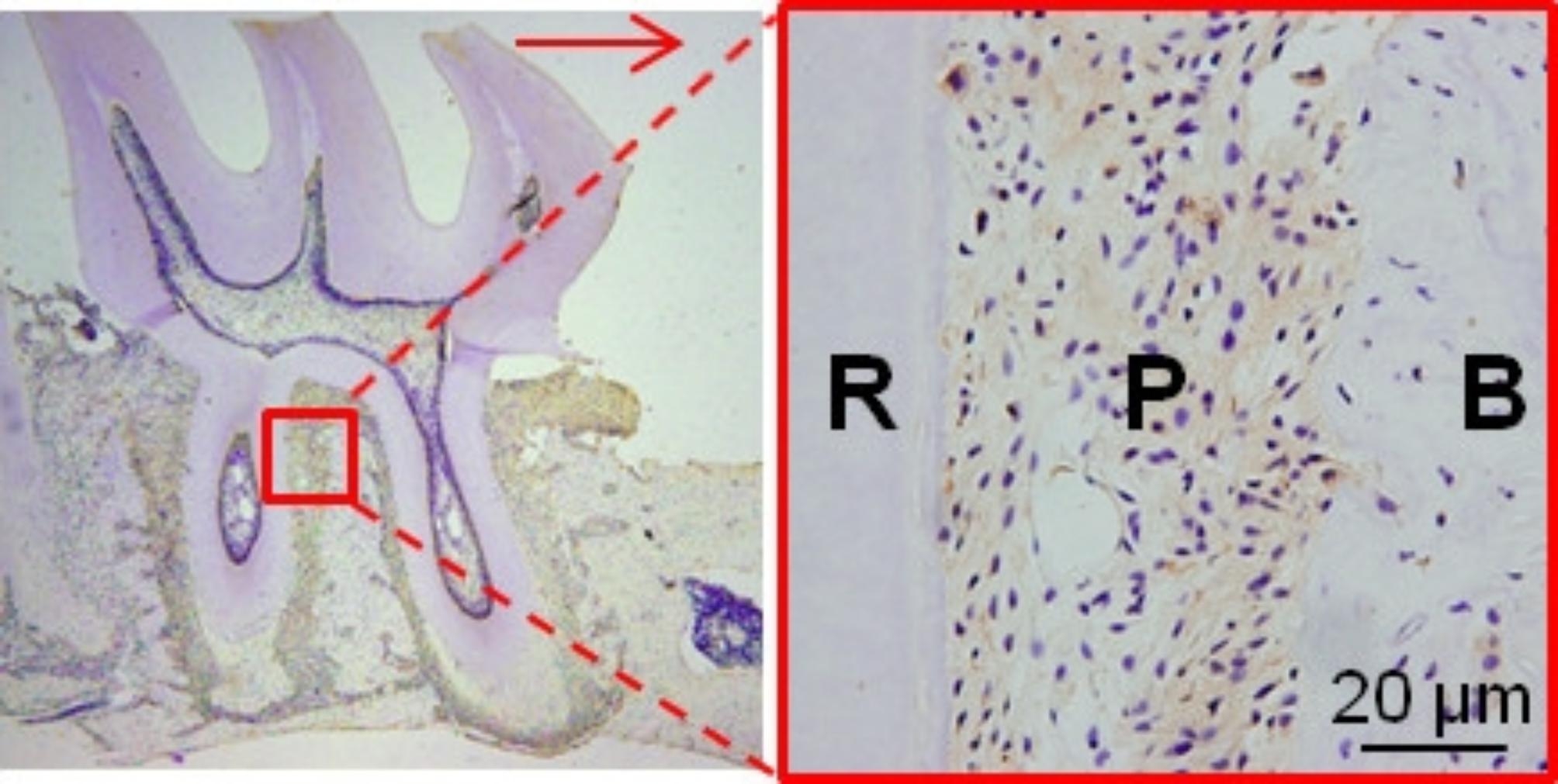
The region selected for immunohistochemical staining. The large boxed area shows high-magnification views of the small boxed area. The red arrow represents the direction of tooth movement. R = distal palatal root; P = periodontal ligament; B = alveolar bone; (Original magnification × 40 on the left and × 400 on the right)

**Fig. 5:**
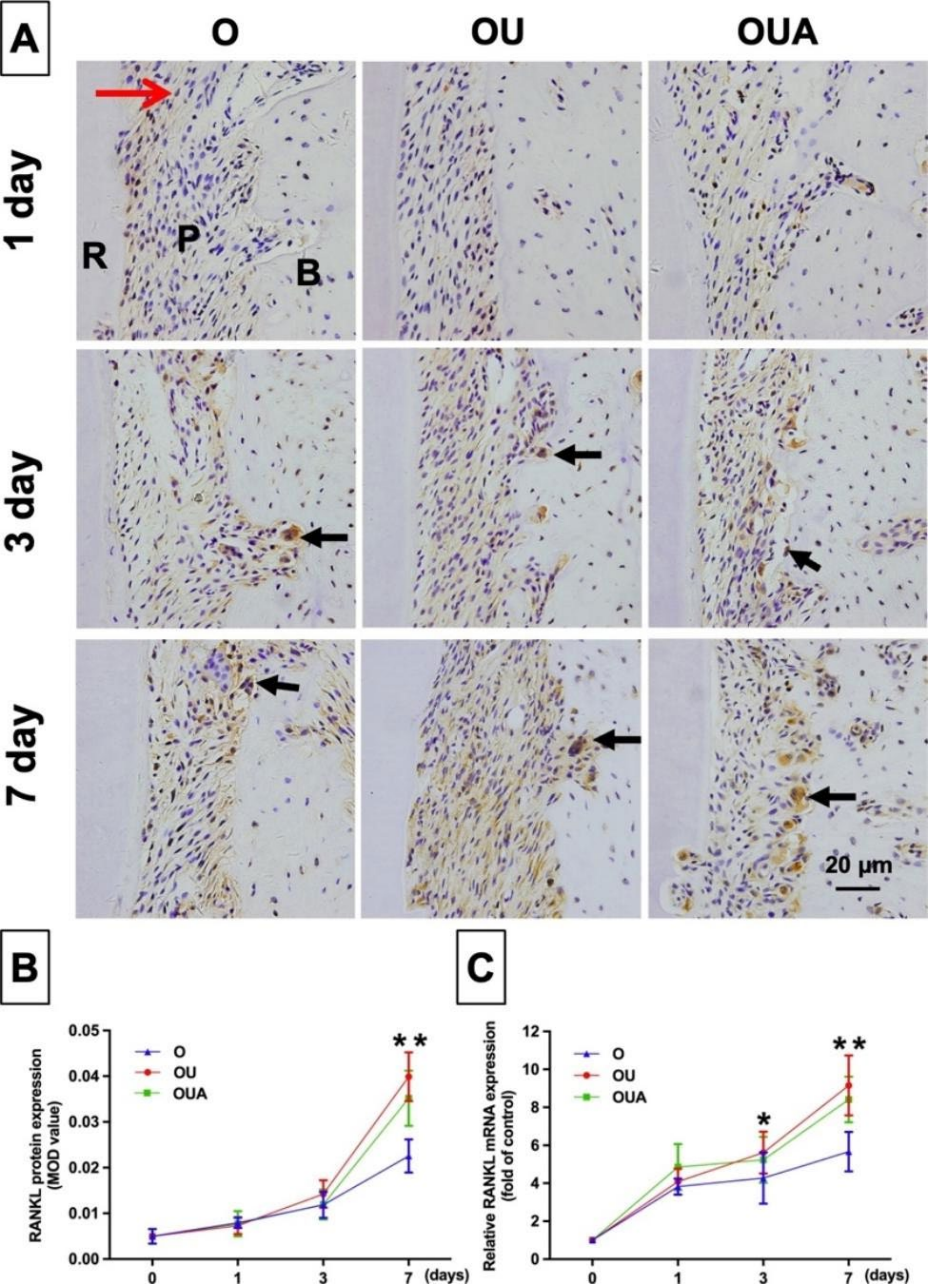
Immunohistochemical staining (**A**), relative protein (**B**), and mRNA (**C**) expression level of RANKL in PDL. The black arrows show a positive expression (× 400). The red arrow represents the direction of tooth movement. R = distal palatal root; P = periodontal ligament; B = alveolar bone. *P < 0.05, OU group versus O group; **P < 0.01, OU group versus O group

**Fig. 6:**
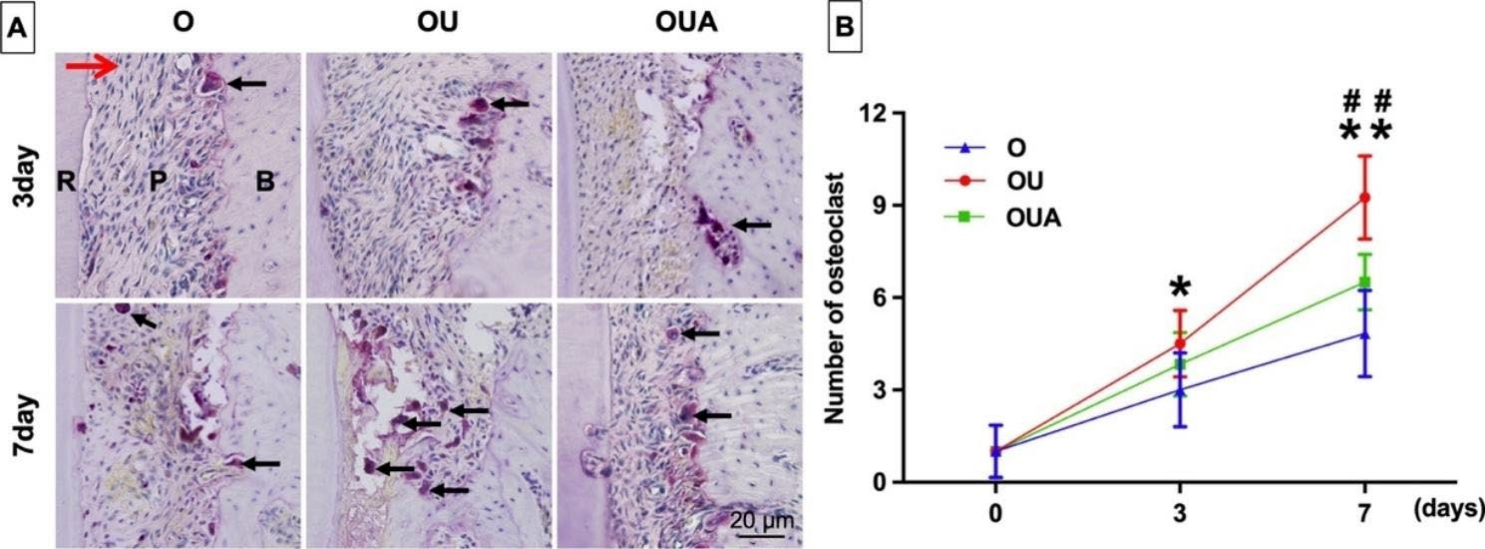
Tartrate-resistant acid phosphatase (TRAP) staining (**A**) and quantitative analysis (**B**) of osteoclasts. The black arrows show the osteoclast (× 400). The red arrow represents the direction of tooth movement. R = distal palatal root; P = periodontal ligament; B = alveolar bone. *P < 0.05, OU group versus O group; ##P < 0.01, OU group versus OUA group; *P < 0.05; **P < 0.01

Orthodontic movement is closely related to osteoclast responses. The selected tissue area for staining and mRNA extraction is shown in Fig. 4. The RANKL produced by osteoblastic can activate osteoclast. Figure 5 showed the expression of RANKL and the results of osteoclastic numbers were shown in Fig. 6.

Based on the q-PCR results, the relative mRNA expression of RANKL increased gradually from day 1 to day 7 (Fig. 5C). On days 3 and 7, the expression of RANKL was significantly higher in the OU group than in the O group (P<0.05). The expression of RANKL was slightly lower in the OUA group than in the OU group. Figure 6 (A, B) shows that there is a similar trend with q-PCR, the expression of RANKL protein increased gradually from day 1 to day 7. The mean optical density value of RANKL was higher in the OU group than in the O group, and the difference was significant on day 7 (P<0.05). Compared with the OU group, there was a reduction in the OUA group, but the difference was not significant.

The number of osteoclasts increased gradually from day 3 to day 7 (Fig. 6). There are significantly more osteoclasts in the OU group than in the O group during these days (P<0.05). The number of osteoclasts in the OUA group was significantly lower than in the OU group on day 7 (P<0.01).

### Bone tissue response to ultrasound

**Fig. 7:**
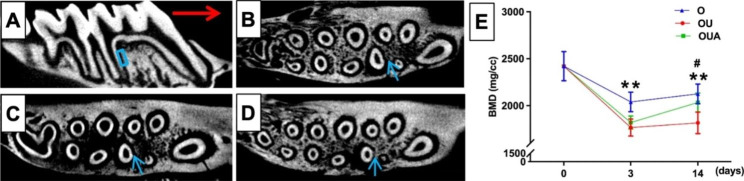
Cross-sections of micro-computed tomography images in three groups: (**B**) O group (**C**) OU group and (**D**) OUA group. (**E**) Bone mineralization density (BMD) on the compression sides. The blue-framed area (representing the compression side) was used for quantitative analysis. The red arrow represents the direction of tooth movement. The blue arrow represents periodontal intersection space for compression sides. *P < 0.05, OU group versus O group; #P < 0.05, OU group versus OUA group; *P < 0.05; **P < 0.01

Orthodontic force is transmitted through periodontal ligaments to the surrounding bone tissue, prompting the reconstruction of the surrounding bone tissue. Figure 7 A showed the direction in the teeth moved. The blue-framed area (representing the compression side) was used for quantitative analysis. As the results of Fig. 7 showed, the density of bone tissue around orthodontic teeth (Fig. 7B) decreased, and in the ultrasound group (Fig. 7C), bone density around the orthodontic teeth and adjacent teeth was lower than that in the orthodontic group. After aspirin administration (Fig. 7D), the density of bone tissue around the teeth was higher than that in the ultrasound group, but lower than the orthodontic group. The Micro-CT image results are consistent with bone mineral density (BMD) analysis results.

**Fig. 8:**
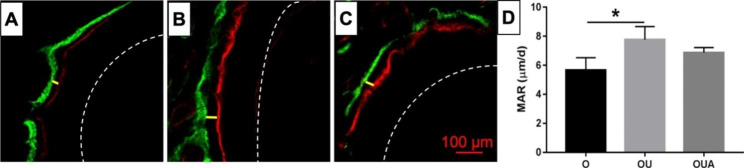
The bone deposition width of Fluorescence-labeled undecalcified bone sectioning and the bone mineral apposition rate (MAR, D). (**A**) O group, (**B**) OU group, and (**C**) OUA group. The yellow lines show the bone apposition width of the tension sides. The dashed lines mark the outline of the dental roots (× 100). *P < 0.05, OU group versus O group

Fluorescent labeling with alizarin red (red) and calcein (green) was used to analyze the bone mineral apposition rate. The results are shown in Fig. 8. The greater the distance (yellow line) between the two curves, the more bone was deposited. The bone mineral apposition rate was greater in the OU group than in the O group (P < 0.05). Compared with the OU group, the bone mineral apposition rate was slightly lower in the OUA group.

## Discussion

In this study, we confirmed that low-frequency high-intensity ultrasound could accelerate orthodontic teeth movement, with ultrasound time (10 s once a day) that was shorter than the 20-minute requirement for low-intensity pulsed ultrasound. High-intensity ultrasound might cause side effects such as skin burns [34, 35]. However, there are also literature studies [36] showing that irradiation within 20s does not produce adverse skin burns, and HIFU treatment is fast and short to fit human patience.

We examined the effect of ultrasound on osteocytes and bone tissue using histomorphology techniques, including TRAP staining, Fluorescence-labeled bone deposition width, and micro-CT according to lectures [37, 38]. The TRAP staining results showed that the OU group had more osteoclasts than the O group. In the previous study [39], corticotomy surgery was discovered to induce osteoclast formation and accelerated orthodontic teeth movement, with a peak in osteoclast formation on the third day. The results of current research showed that the number of osteoclasts increased gradually from the 3rd to the 7th day. Compared with corticotomy, ultrasound may be a more progressive method of accelerating orthodontic teeth movement.

During orthodontic teeth movement, the Micro-CT results showed that bone resorbed on the pressure side. Following ultrasound treatment, the bone mineralization density of the pressure side decreased, which is consistent with the reported increase in osteoclasts. The micro-CT results showed that the bone mineralization density in OU group was lower than that in O group. The number of osteoclasts increased after ultrasound and bone tissue was absorbed on the pressure side. Osteoblasts activated are the necessary factors for the expression of specific mediators, such as RANKL, for osteoclast formation and the initiation of bone resorption. Orthodontic tooth movement is the process of bone tissue modeling and remodeling [38]. The results of bone mineral apposition rate showed the bone formation of tension side. Frost [40] proposed the “mechanical threshold theory,” which states that bone tissue is dynamically balanced between bone absorption and bone formation in normal physiological conditions. Mechanical signals can be translated into biological signals that regulate bone tissue remodeling when they are activated by machinery [41]. After ultrasound in this research, bone tissue remodeling increased in the process of tooth movement.

Aspirin can always use to relieve pain [42]. The results of OUA group showed that it attenuates the effects of ultrasound. The results of previous studies also showed that non-steroidal anti-inflammatory drugs could reduce orthodontic tooth movement [29, 30, 43]. However, our study also showed that although rats were given aspirin after ultrasound, there was also an increase in orthodontic tooth movement of OUA group. RANKL is expressed as membrane-bound ligands by a variety of cell types, such as osteoblasts, fibroblasts, bone marrow stromal cells. When RANKL binds to homologous RANK receptors on the surface of preosteoclasts, causing them to differentiate into multinucleated mature osteoclasts. At the tissue level, osteoclasts attach to the surface of the bone and subsequently absorb the bone. Meantime, aspirin maybe reduced the number of osteoclasts by influencing the RANKL/RANK signaling pathway, thereby reducing tooth movement distance. Ultrasound may accelerate tooth movement by facilitating RANKL/RANK signaling pathways. This intervention is an inspiration for orthodontics of teeth with difficulty moving.

## Conclusions

The non-invasive and rapid Low-Frequency High-Intensity Ultrasound (LFHIU, 44 W/cm^2^, 28 kHz) can encourage tooth movement. Ultrasound therapy promoted tooth mobility, expedited bone tissue absorption, and boosted osteoclast activity. LFHIU may be a beneficial treatment for orthodontic teeth that are difficult or slow to move. Different frequencies or intensities of ultrasound may have different effects on tissue, so more comfortable and effective ultrasound parameters need to be explored.

## Data Availability

The datasets generated and analyzed during the current study are not publicly available but are available from the corresponding author on reasonable request.

## Abbreviations

O group: Orthodontic Group
OU group: Ultrasound-treated orthodontic Group
OUA group: Ultrasound-treated orthodontic with aspirin gavage Group
LFHIU: Low-frequency high-intensity ultrasound
OTM: Orthodontic teeth movement
RANKL: Receptor activator of nuclear factor kappa-B ligand
MOD: Mean optical density
BMD: Bone mineral density
TRAP: Tartrate-resistant acid phosphatase
MAR: Mineral apposition rate
